# Impact of malaria and helminth infections on immunogenicity of the human papillomavirus-16/18 AS04-adjuvanted vaccine in Tanzania^☆^^☆☆^

**DOI:** 10.1016/j.vaccine.2013.11.061

**Published:** 2014-01-23

**Authors:** Joelle Brown, Kathy Baisley, Bazil Kavishe, John Changalucha, Aura Andreasen, Philippe Mayaud, Balthazar Gumodoka, Saidi Kapiga, Richard Hayes, Deborah Watson-Jones

**Affiliations:** a University of California, San Francisco, Department of Epidemiology and Biostatistics, San Francisco, CA, USA; b University of California, Los Angeles, Department of Epidemiology, Los Angeles, CA, USA; c Faculty of Infectious and Tropical Diseases, London School of Hygiene and Tropical Medicine, London, UK; d Mwanza Intervention Trials Unit, National Institute for Medical Research, Mwanza, Tanzania; e National Institute for Medical Research, Isamilo, Mwanza, Tanzania; f Faculty of Epidemiology and Population Health, London School of Hygiene and Tropical Medicine, London, UK; g Bugando Medical Centre, Mwanza, Tanzania

**Keywords:** Human papillomavirus, HPV, Vaccine, Immunogenicity, Malaria, Helminth, Parasitic infection, Tanzania, Sub-Saharan Africa

## Abstract

**Background:**

Endemic malaria and helminth infections in sub-Saharan Africa can act as immunological modulators and impact responses to standard immunizations. We conducted a cohort study to measure the influence of malaria and helminth infections on the immunogenicity of the bivalent HPV-16/18 vaccine.

**Methods:**

We evaluated the association between malaria and helminth infections, and HPV-16/18 antibody responses among 298 Tanzanian females aged 10–25 years enrolled in a randomized controlled trial of the HPV-16/18 vaccine. Malaria parasitaemia was diagnosed by examination of blood smears, and helminth infections were diagnosed by examination of urine and stool samples, respectively. Geometric mean antibody titres (GMT) against HPV-16/18 antibodies were measured by enzyme-linked immunosorbent assay.

**Results:**

Parasitic infections were common; one-third (30.4%) of participants had a helminth infection and 10.2% had malaria parasitaemia. Overall, the vaccine induced high HPV-16/18 GMTs, and there was no evidence of a reduction in HPV-16 or HPV-18 GMT at Month 7 or Month 12 follow-up visits among participants with helminths or malaria. There was some evidence that participants with malaria had increased GMTs compared to those without malaria.

**Conclusions:**

The data show high HPV immunogenicity regardless of the presence of malaria and helminth infections. The mechanism and significance for the increase in GMT in those with malaria is unknown.

## Highlights

• First study to measure impact of malaria and helminth infection on HPV vaccine response.
• Study nested within a clinical trial of the HPV-16/18 vaccine in Tanzania.
• High prevalence of parasitic infections among Tanzanian girls and young women.
• HPV vaccine highly immunogenic regardless of the presence of malaria and helminths.
• Participants with malaria had increased vaccine response compared to those without.

## 1 Introduction

Human papillomavirus (HPV) genotypes 16 and 18 are estimated to cause 70% of cervical cancers worldwide [1]. Over 85% of the global burden of cervical cancer occurs in developing countries and Tanzania reports one of highest rates of cervical cancer in Africa [2]. Potent, durable HPV vaccine efficacy will be essential if the vaccine is introduced for the control of cervical cancer. Endemic infections in sub-Saharan Africa, such as malaria and helminth infections, act as immunological modulators, and have been found to adversely impact immune response to standard immunizations, such as antituberculosis vaccine bacillus Calmette–Guerin (BCG), typhoid fever, tetanus and polio vaccines [3], [4], [5], [6], [7], [8], [9]. Studies to evaluate the effect of HPV vaccines in populations whose immunological system may be challenged by multiple co-infections such as malaria and helminth infections are needed [10], [11]. We conducted a study to measure the influence of malaria parasitaemia and helminth infection on the immunogenicity of HPV-16/18 vaccine (GlaxoSmithKline (GSK) Biologicals SA). This study was nested within a cohort recruited for a Phase IIIb immunogenicity and safety trial of the HPV-16/18 vaccine (the HPV 021 trial) conducted in Tanzania and Senegal among HIV-negative girls and young women aged 10–25 years [12].

## 2 Methods

The HPV 021 trial (NCT00481767) and the malaria/helminth study were conducted from October 2007 to July 2010 in Mwanza, Tanzania, one of the two participating HPV-021 trial centres. GSK Biologicals was the funding source for the studies. Both studies were approved by the ethics committees of the National Institute for Medical Research (NIMR), Tanzania and the London School of Hygiene & Tropical Medicine (LSHTM), United Kingdom. The helminth/malaria study was registered under ControlledTrials.com (ISRCTN90378590).

The HPV 021 trial was a double-blind, randomized, placebo-controlled phase IIIb trial. Eligible participants were randomly assigned (2:1) to receive either three doses of HPV-16/18 AS04-adjuvanted vaccine (vaccine group) or Al(OH)3 (placebo group) at 0,1 and 6 months. After enrolment (Month 0), participants returned to the clinic at Months 1, 2, 4, 6, 7, 8, 10 and 12 for follow-up visit procedures. Participants were tested for malaria and helminth infections at the Month 7 visit, one month after the scheduled vaccine dose 3. In addition, participants could attend government health services for investigation and management of any illnesses between booked study visits. A record was kept of investigations and treatments given through these other health services.

The primary objective of this analysis was to evaluate the association of malaria parasitaemia and helminth infection with antibody responses against HPV-16 and HPV-18 one month (Month 7) and six months (Month 12) after the last scheduled vaccine dose in African females aged 10–25 years.

Potential participants were recruited from schools, colleges and family planning clinics in Mwanza, and invited to attend a screening visit for eligibility approximately one month prior to enrolment. Prior to screening, informed consent was obtained from participants aged 18–25 years. For participants aged 10–17 years, we sought consent from a parent or legally authorized representative, as well as assent from the participant. Participants were eligible for enrolment if they were aged 10–25 years at the time of first vaccination, HIV negative, not pregnant, had not had more than six lifetime sexual partners, were free of obvious health problems as established by medical history and examination, had no history of neurologic disorders and were willing to use contraception or to abstain from sex if sexually active for 30 days prior to vaccination and for two months after completion of vaccination. The enrolment was age-stratified, with one-third of participants in the 10–14 years age-stratum and the remainder in the 15–25 years age-stratum.

Study procedures for the HPV 021 trial have been described in detail elsewhere [12]. In brief, the HPV vaccine and placebo were administered intramuscularly into the deltoid muscle of the non-dominant arm at the Month 0 visit and again at Month 1 and Month 6 visits. Sociodemographic characteristics were collected at Month 0 in face-to-face interviews using standardized questionnaires. Blood samples were collected at Months 0, 2, 7 and 12 to evaluate antibody responses against HPV-16 and HPV-18 by enzyme-linked immunosorbent assay (ELISA). In order to test for helminth infection and malaria parasitaemia at Month 7, participants provided (i) a blood sample for the diagnosis of malaria, (ii) a first void urine sample for the diagnosis of *Schistosoma haematobium* and (iii) three separate stool samples (during the week following the Month 7 visit) for the diagnosis of *Schistosoma mansoni, Ancylostoma duodenale* (hookworm), *Strongyloides stercoralis, Ascaris lumbricoides, Trichuris trichiura and Taenia* spp. Participants who tested positive for malaria or helminth infections were provided with treatment by study clinicians at a subsequent study visit.

### 2.1 Laboratory testing

#### 2.1.1 Malaria

Pairs of thick and thin peripheral blood films from each patient were stained with Giemsa stain in Mwanza, and examined by light microscopy at NIMR in Mwanza, and confirmed at LSHTM. Each thick film was scanned under oil immersion for at least 5 min and the presence of asexual malaria parasites or sexual gametocytes was recorded. Where parasites were seen, the number per 200 white blood cells (WBC) on the thick film was counted and multiplied by 40 to give number of parasites per microliter (parasite density, assuming 8000 WBC per μL as per World Health Organization recommendations for Africa) [13]. In thin films, parasite detection (where possible) and species confirmation was done by scanning for a similar duration.

#### 2.1.2 Helminths

A 10 mL aliquot from each urine sample was filtered through 25 mm, 12 μm Millipore filters on Swinnex filter holders. After filtration, the filter was placed onto a glass slide using blunt forceps adding a drop of saline and a glass coverslip. The filter was then examined at the NIMR laboratory under light microscopy for the eggs of *S. haematobium.*

Stool samples were examined at the NIMR laboratory for quantitative egg counts for *S. mansoni*, hookworm, *S stercoralis, A. lumbricoides, T. trichiura* and *Taenia* spp. using the Kato-Katz method [14], [15]. The stool samples were first homogenised by passing through a sieve, and then a 41.7 mg template was used. The faecal portion was covered with a cellophane square that had been soaked in malachite green and glycerol. The sample was examined immediately and then again after 24 h. Eggs were counted and expressed as eggs per gram of faeces. For quality control, a random sample of 10% of positive and negative stool slides were sent to the Uganda Virus Research Institute/Medical Research Council laboratories in Entebbe for repeat Kato-Katz testing.

In addition, charcoal culture was used to confirm *S. stercoralis* in a subset of samples. Approximately 50 mg of unfixed fresh faeces were mixed with distilled water in a 20 mL universal tube [16]. To this suspension an equal volume of granulated hardwood charcoal was added. After mixing, the suspension was placed over a wet disc of filter paper in a petri dish and stored in the dark at room temperature. The petri dishes were observed daily for the presence of larvae for a week under a dissection microscope, adding water to the filter paper as needed.

#### 2.1.3 HPV immunogenicity

As part of the HPV 021 trial, serological assays for immunogenicity were performed at a GSK laboratory in Belgium. ELISA was used to determine antibodies to HPV-16 and HPV-18 as described previously [17]. As there are no established immunological correlates of protection for HPV-16 or HPV-18, immunogenicity was determined in terms of seroconversion rates and geometric mean antibody titres (GMTs). Seropositivity was defined as an antibody titre greater than or equal to the assay threshold of 8 ELISA units (EU)/mL for HPV-16 and 7 EU/mL for HPV-18 [17].

### 2.2 Analyses

Data were double entered and verified in DMSys^®^ (SigmaSoft International) and analysed using STATA11.0 (StataCorp LP; College Station, Texas, USA). Sociodemographic characteristics of participants attending the Month 7 visit were tabulated by infection status and overall. The prevalence of malaria parasitaemia and each helminth infection at Month 7 was tabulated by age group and overall. Helminth infection intensities were classified into light, moderate and heavy, according to WHO guidelines [18]. For each individual, the arithmetic mean of the helminth species-specific egg counts from the Kato-Katz thick stool smears was calculated and multiplied by 24, to obtain the eggs per gram of faeces (EPG). The upper limits of light and moderate infections were 100 and 400 EPG for *S. mansoni*; 2000 and 4000 EPG for hookworm; 1000 and 10,000 EPG for *T. trichiura* and 5000 and 50,000 EPG for *A. lumbricoides*, respectively. For *S. haematobium*, egg counts from urine were classified into two categories only, light (<50 eggs/10 mL of urine) and heavy (≥50 eggs/10 mL of urine or visible haematuria). There were too few participants in the vaccine-arm who were co-infected with both malaria and helminth infections (*n* = 8), or multiple helminth infections (*n* = 6) to examine the relationship between co-infection and HPV immunogenicity.

Because the anti-HPV-16 and HPV-18 IgG antibody concentrations showed skewed distributions, HPV antibody results were transformed as log_10_ (IgG concentration). Geometric mean titres (GMT, EU/mL) and 95% confidence intervals (CI) were calculated.

The analysis of HPV vaccine antibody response, and malaria and helminth infection was restricted to participants in the vaccine-arm who attended the Month 7 visit (*n* = 195) or the Month 12 visit (*n* = 196) and had immunogenicity results. Box plots were used to graphically examine the distribution of raw antibody responses by malaria and helminth infection status. Linear regression was used to compare mean log-transformed IgG antibody between participants with and without any helminth infection, and with and without malaria. Regression coefficients and confidence limits were back-transformed to express results as ratios of geometric means (GMR). These analyses controlled for potential confounding by age of participants, and number of vaccine doses received. Analyses of malaria and HPV vaccine antibody response controlled for presence of any helminth infection. Similarly, the analyses of helminth infection and HPV vaccine antibody response controlled for malaria parasitaemia. There were insufficient data to examine associations with specific helminth infections.

## 3 Results

### 3.1 Cohort screening, enrolment and follow-up

In total 587 participants attended the screening visit, and 334 were enrolled in the HPV 021 trial. Of these, 221 participants were randomized to the vaccination arm and 113 to the placebo-arm. Overall, 298 (89%) participants attended the Month 7 visit (90 and 88% in the vaccine and placebo arms, respectively) and 308 (92%) attended the Month 12 visit (93 and 90% in the vaccine and placebo arms, respectively). The most common reason for discontinuation was withdrawal of consent (4%). The majority (96%) of participants received all three vaccine or placebo doses (Table 1); number of doses received did not differ substantially between participants in the vaccine and placebo arms or between those with and without malaria and/or helminth infections (Table 1) in either trial arm.

**Table 1:** Characteristics of cohort attending for Month 7 visit.

	By infection status^a^ (*N* = 273)			
	No infection (*N* = 169) *n* (%)	Any helminth^b^ (*N* = 86) *n* (%)	Malaria^b^ (*N* = 29) *n* (%)	Total^c^ (*N* = 298) *n* (%)
Age group (years)				
10–14	62 (36.7)	25 (29.1)	14 (48.3)	107 (35.9)
15–19	74 (43.8)	48 (55.8)	10 (34.5)	138 (46.3)
20–25	33 (19.5)	13 (15.1)	5 (17.2)	53 (17.8)

Tribe^d^				
Sukuma	57 (33.9)	30 (34.9)	9 (31.0)	97 (32.7)
Non-sukuma	111 (66.1)	56 (65.1)	20 (69.0)	200 (67.3)

Religion^d^				
Catholic	79 (47.3)	41 (47.7)	15 (51.7)	137 (46.3)
Other christian	48 (28.7)	22 (25.6)	7 (24.1)	81 (27.4)
Muslim	40 (24.0)	23 (26.7)	7 (24.1)	78 (26.4)

Education level^d^				
Less than primary	47 (28.0)	27 (31.4)	11 (37.9)	87 (29.3)
Primary	39 (23.2)	20 (23.3)	7 (24.1)	70 (23.6)
Secondary	77 (45.8)	37 (43.0)	11 (37.9)	129 (43.4)
Above secondary	5 (3.0)	2 (2.3)	0 (-)	11 (3.7)

Marital status^d^				
Single	149 (88.7)	75 (87.2)	25 (86.2)	265 (89.2)
Married	19 (11.3)	11 (12.8)	3 (10.3)	31 (10.4)
Divorced/separated	0	0	1 (3.5)	1 (0.3)

Occupation^d^				
Student	137 (82.5)	67 (77.9)	24 (82.8)	243 (82.4)
Manual/clerical/other	12 (7.2)	7 (8.1)	3 (10.3)	21 (7.1)
Housewife/unemployed	17 (10.2)	12 (14.0)	2 (6.9)	31 (10.5)

Housing construction^d^				
Cement blocks	68 (41.0)	27 (31.4)	10 (34.5)	108 (36.6)
Mud bricks	64 (38.6)	34 (39.5)	12 (41.4)	118 (40.0)
Burnt bricks	26 (15.7)	14 (16.3)	6 (20.7)	50 (16.9)
Other	8 (4.8)	11 (12.8)	1 (3.5)	19 (6.4)

Vaccine doses received				
Three	160 (94.7)	85 (98.8)	29 (100)	287 (96.3)
Less than three	9 (5.3)	1 (1.2)	0	11 (3.7)

a Among 273 participants with complete data on all infections.

b Includes 11 participants who were positive for both helminth and malaria infection.

c Among 298 participants who attended the 7 month visit.

d Missing data on tribe, education and marital status for 1 participant. Missing data on religion for 2 participants. Missing data on occupation and housing construction for 3 participants.

All participants were of African origin and were HIV-seronegative at baseline. The median age of participants was 18 years (IQR = 13–19). More than three-quarters of participants (82%) were currently students. Most (89%) participants were single. Approximately one-third (37%) of participants lived in houses constructed from cement blocks, and 40% lived in homes constructed from mud bricks (Table 1). As previously reported, sociodemographic characteristics did not differ by vaccine-arm [12].

### 3.2 Prevalence of malaria and helminths at Month 7

At Month 7, approximately one-third (38.1%) of participants tested positive for either malaria parasitaemia or helminth infection. The prevalence of malaria parasitaemia in the entire cohort was 10.2% (Table 2) and in the vaccinated cohort was 10.5%. The prevalence of any helminth infection was 30.4% in the entire cohort (Table 2), and 31.6% in the vaccinated cohort. *S. mansoni* was the most commonly detected helminth, found in one-quarter of participants (24.0%), followed by hookworm (5.7%). *S. haematobium* was rare; only two (0.7%) participants tested positive. The prevalence of malaria parasitaemia was somewhat higher in younger participants (Table 2), although there was not strong evidence of a difference (*p* = 0.24).

**Table 2:** Prevalence^a^ of helminths and malaria infection at Month 7, by age group and overall.

	10–14 years (*N* = 107) *n* (%)	15–19 years (*N* = 138) *n* (%)	20–25 years (*N* = 53) *n* (%)	All ages (*N* = 298) *n* (%)
*S. mansoni*^b^	20 (19.6)	42 (32.1)	6 (12.0)	68 (24.0)
Hookworm^b^	2 (2.0)	7 (5.3)	7 (14.0)	16 (5.7)
*S. stercoralis*^b^	0	0	0	0
*A. lumbricoides*^b^	2 (2.0)	0	0	2 (0.7)
*T. trichiura*^b^	2 (2.0)	1 (0.8)	3 (6.0)	6 (2.1)
*Taenia* spp.^b^	0	0	1 (2.0)	1 (0.4)
*S. haematobium*	1 (0.9)	1 (0.7)	0	2 (0.7)
Any helminth^c^	25 (24.5)	48 (36.6)	13 (26.0)	86 (30.4)
Malaria^d^	14 (14.1)	10 (7.5)	5 (9.6)	29 (10.2)

Number of infections^e^				
None	62 (64.6)	74 (57.8)	33 (67.4)	169 (61.9)
1	27 (28.1)	47 (36.7)	10 (20.4)	84 (30.8)
2	6 (6.3)	7 (5.5)	6 (12.2)	19 (7.0)
3	1 (1.0)	0	0	1 (0.4)

a Prevalence of each infection is among those without missing data for that organism.

b Missing helminth results for 5 participants in 10–14 years age group, 7 in the 15–19 years age group and 3 in the 20–25 years age group.

c Among 283 participants with complete data on all helminths.

d Missing malaria results from 8 participants in the 10–14 years age group, 4 particpants in the 15–19 years age group and 1 participant in the 20–25 years age group.

e Among 273 participants with complete data on all infections.

Three quarters (77.9%) of *S. mansoni* infections were light infections, 17.6% were moderate and 4.4% were heavy. Of the two *S. haematobium* infections, one was light and one was heavy. All (100%) of the hookworm, *A. lumbricoides, T. trichiura and Taenia* spp. infections were categorized as light infections.

### 3.3 Geometric mean titres for HPV-16/18 antibody response

As previously reported, all initially seronegative participants in the vaccinated cohort seroconverted for anti-HPV-16 and -18 antibodies, and remained seropositive up to Month 7. At Month 12, all initially seronegative participants in the vaccine group remained seropositive for anti-HPV-16, and all except one (13-year-old girl) remained seropositive for anti-HPV-18 [12]. Four participants had missing antibody results at Month 7, but were seropositve for anti-HPV-16 and -18 antibodies at Month 12.

HPV immunogenicity was high at Month 7 and Month 12. Among the vaccinated cohort who attended the Month 7 visit and had antibody results (*n* = 195), the GMT HPV-16 antibody response at Month 7 was 10,786 EU/mL (95% CI 9126–12,747), and the GMT HPV-18 antibody response was 3701 EU/mL (95% CI 3156–4340) (Table 3). As previously reported, HPV-16/18 serostatus at enrolment (prior to vaccination) did not influence GMTs at Month 7 or Month 12 [12]. GMT HPV-16 and HPV-18 antibody responses at Month 7 were at least 2 fold higher in 10–14-year-olds (19,374 EU/mL, 95% CI 16,600–22,611 and 5723 EU/mL, 95% CI 4790–6839, respectively) than in 15–25-year-olds (7770 EU/mL, 95% CI 6188–9755 and 2900 EU/mL, 95% CI 2333–3605, respectively, *P* < 0.001).

**Table 3:** Antibody responses at Month 7 and at Month 12 in vaccinated participants by helminth infection and malaria infection status.

	*N*	Geometric mean titre (EU/mL) (95% CI)	Unadjusted geometric mean ratio (95% CI)	Adjusted geometric mean ratio^a^ (95% CI)
Month 7				
*HPV-16 IgG*				
Overall	195	10786 (9126–12747)	–	–
Any helminth				
No	126	10492 (8445–13036)	*P* = 0.27	*P* > 0.99
Yes	60	12761 (10269–15857)	1.22 (0.86–1.72)	1.00 (0.77–1.29)
Intensity of helminth infection				
None	126	10492 (8445–13036)	*P* = 0.48	*P* = 0.72
Light	50	12363 (9936–15383)	1.18 (0.81–1.71)	0.96 (0.73–1.26)
Moderate/heavy	10	14946 (6442–34679)	1.42 (0.69–2.95)	1.20 (0.71–2.03)
Malaria				
No	166	9750 (8082–11761)	*P* = 0.01	*P* = 0.05
Yes	20	20357 (14430–28720)	2.09 (1.20–3.63)	1.47 (1.00–2.18)
*HPV-18 IgG*				
Overall	195	3701 (3156–4340)	–	–
Any helminth				
No	126	3513 (2880–4285)	*P* = 0.19	*P* = 0.64
Yes	60	4392 (3418–5643)	1.25 (0.90–1.75)	1.06 (0.82–1.38)
Intensity of helminth infection				
None	126	3513 (2880–4285)	*P* = 0.26	*P* = 0.35
Light	50	4129 (3162–5393)	1.18 (0.82–1.68)	1.00 (0.75–1.32)
Moderate/heavy	10	5973 (2689–13268)	1.70 (0.84–3.42)	1.46 (0.85–2.51)
Malaria				
No	166	3434 (2873–4104)	*P* = 0.07	*P* = 0.42
Yes	20	5648 (3736–8538)	1.64 (0.97–2.80)	1.18 (0.79–1.76)

Month 12				
*HPV-16 IgG*				
Overall	196	2656 (2246–3140)	–	–
Any helminth				
No	129	2613 (2124–3215)	*P* = 0.64	*P* = 0.70
Yes	59	2843 (2171–3723)	1.09 (0.76–1.55)	0.94 (0.67–1.31)
Intensity of helminth infection				
None	129	2617 (2129–3217)	*P* = 0.67	*P* = 0.70
Light	49	2994 (2301–3895)	1.14 (0.78–1.67)	0.98 (0.69–1.40)
Moderate/heavy	10	2218 (745–6600)	0.85 (0.40–1.77)	0.75 (0.38–1.48)
Malaria				
No	167	2461 (2039–2971)	*P* = 0.05	*P* = 0.16
Yes	20	4335 (2890–6502)	1.76 (1.01–3.08)	1.43 (0.86–2.37)
*HPV-18 IgG*				
Overall	196	986 (834–1166)	–	–
Any helminth				
No	129	970 (781–1205)	*P* = 0.71	*P* = 0.89
Yes	59	1038 (802–1344)	1.07 (0.74–1.54)	0.98 (0.69–1.38)
Intensity of helminth infection				
None	129	973 (784–1207)	*P* = 0.83	*P* = 0.85
Light	49	1076 (806–1436)	1.11 (0.75–1.63)	1.01 (0.70–1.47)
Moderate/heavy	10	880 (453–1712)	0.90 (0.42–1.93)	0.82 (0.41–1.66)
Malaria				
No	167	952 (787–1151)	*P* = 0.59	*P* = 0.79
Yes	20	1109 (764–1609)	1.16 (0.66–2.05)	0.93 (0.55–1.58)

a Geometric mean ratio (GMR) for helminth infection adjusted for participant age, number of vaccine doses and malaria infection. GMR for malaria infection adjusted for age, number of vaccine doses and any helminth infection.

Antibody responses to HPV-16/18 among 107 vaccine-arm participants without helminths or malaria parasitaemia were high. The GMT HPV-16 antibody response among helminth and malaria uninfected 10–14-year-olds at Month 7 (*N* = 40) was 18,248 EU/mL (95% CI 14,742–22,587), and for 15–25-year-olds (*N* = 67) was 6493 EU/mL (95% CI 4606–9153). Similarly, the GMT HPV-18 antibody response among helminth and malaria uninfected 10–14-year-olds at Month 7 was 5255 EU/mL (95% CI 4109–6720), and for 15–25-year-olds was 2479 EU/mL (95% CI 1807–3399).

**Fig. 1:**
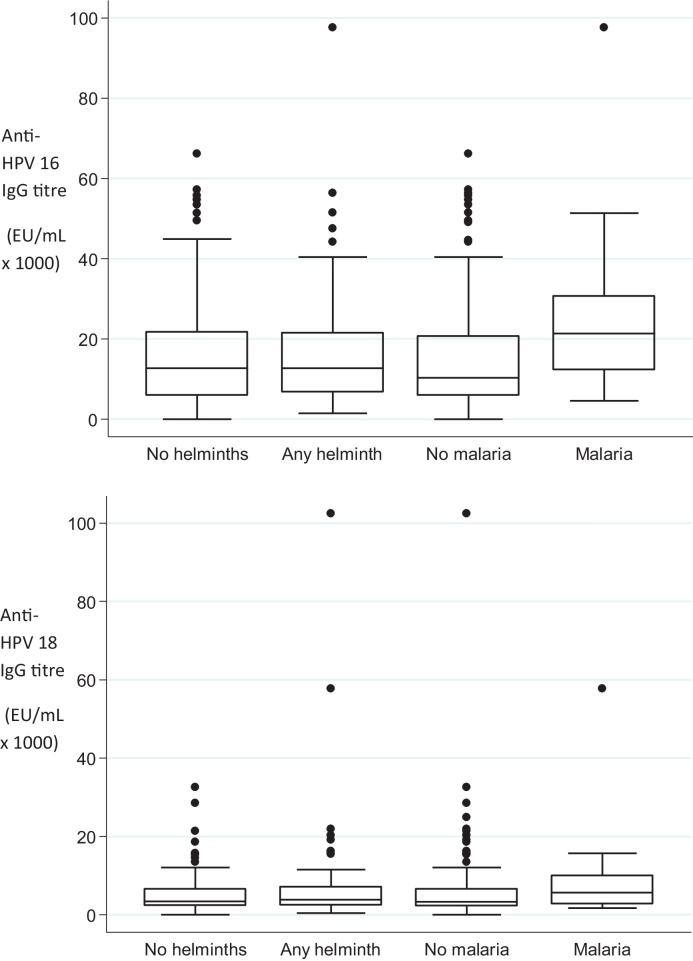
Anti-HPV-16 (top) and HPV-18 (bottom) titre at 7 months after first dose of HPV vaccine in Tanzanian females aged 10–25 years, by helminth and malaria infection status. The central line represents the median; boxes represent 75th and 25th centiles; whiskers represent upper and lower adjacent values and dots represent outside values.

There was some evidence that participants with malaria parasitaemia at Month 7 had a higher GMT HPV-16 and HPV-18 antibody response (Table 3; Fig. 1). After controlling for age, number of vaccine doses received, and any helminth infection, participants with evidence of malaria had a roughly 1.5 fold higher HPV-16 GMT than participants without malaria (adjusted geometric mean ratio (GMR) = 1.47, 95% CI 1.00–2.18, *P* = 0.05). Participants with malaria parasites had a 1.2 fold higher GMT HPV-18 antibody response at Month 7 compared to participants without malaria (adjusted GMR = 1.18, 95% CI 0.79–1.76, *P* = 0.42).

At the Month 12 visit, there was also some evidence that the HPV-16 GMT antibody response was higher among participants with malaria parasitaemia at Month 7, adjusting for age, number of vaccine doses received, and any helminth infection (adjusted GMR = 1.43, 95% CI 0.86–2.37, *P* = 0.16) (Table 3). There was no evidence of a difference in HPV-18 GMT antibody response at Month 12 between participants with malaria parasitaemia at Month 7 and those without (adjusted GMR = 0.93, 95% CI 0.55–1.58, *P* = 0.79) (Table 3).

At Month 7 and Month 12, GMT antibody responses were similar in participants with and without helminth infections (Table 3). The GMR for HPV-16 antibody response at Month 7, comparing participants with and without helminth infection, was 1.00 (95% CI 0.77–1.29, *P* > 0.99), after controlling for age, number of vaccine doses received and malaria parasitaemia (Table 3; Fig. 1). The adjusted GMR for HPV-18 antibody response comparing participants with and without helminth infection was 1.06 (95% CI 0.82–1.38, *P* = 0.64). Similar results were seen at Month 12. Although mean antibody response was highest in participants with higher intensity helminth infections, there was no evidence of a signficant difference (Table 3).

## 4 Discussion

This is the first study to examine the effect of malaria and helminth infections on HPV vaccine antibody responses. The incidence of cervical cancer is extremely high in many countries in sub-Saharan Africa which are considering the implementation of HPV vaccination as a cervical cancer control strategy but which also have a high prevalence of endemic malaria and helminth infections. These infections can impact immune responses to vaccinations [3], [4], [5], [6], [7], [8], [9]. Reassuringly, we found no negative impact on the immune response to the HPV-16/18 vaccine in the presence of these infections. The HPV-16/18 vaccine was highly immunogenic, especially in younger girls, as previously observed [19].

We observed some evidence of an association between malaria parasitaemia and a higher antibody response to the HPV-16/18 vaccine, which persisted adjusting for age. This association appeared weaker at Month 12 than Month 7 perhaps because there was a longer interval between the timing of the malaria and helminth tests and the antibody data. There was no observed effect of helminth infection, or intensity of helminth infection, on HPV-16/18 antibody response. The mechanism and significance of the increase in HPV-16/18 GMTs among malaria infected individuals is unclear. It is possible that malaria may induce a broader spectrum antibody response than helminths, which may potentiate the immune response to the HPV vaccine. We were unable to assess whether this observation was sustained beyond 12 months of follow-up.

As in all observational studies, these findings may be distorted by unmeasured confounders. We attempted to control for potential confounding by age and number of vaccine doses received, which produced little change in the effect estimates. This study also had a small sample size, and a relatively small number of participants with helminth and malaria infections. Results should therefore be interpreted with caution. Sensitivity of the Kato-Katz method in diagnosing helminth infections is relatively low, although we attempted to increase the sensitivity by collecting 3 stool samples from each participant [20], [21]. Finally, infection diagnosed at one point during follow-up will not be representative of infection status at the time that earlier vaccine doses were administered. We were therefore unable to measure the effect of earlier infections on the response to the first and second doses of vaccine.

Both animal and human studies indicate that parasitic infections can impair long-term responses to vaccination [10], [22]. Although our results are encouraging up to one year post-vaccination, because of the short-term nature of this study, our data do not allow us to evaluate whether untreated malaria or helminth infections, repeated infections or co-infections may impair long-term responses to the HPV vaccine. Longer-term follow-up of vaccinated cohorts and repeated cross-sectional surveys to assess antibody response and helminth/malaria infections in communities are warranted.

In summary, we found high HPV immunogenicity regardless of the presence of malaria and helminth infections among young girls and women in Tanzania. There was some evidence of enhanced antibody titres to HPV vaccine genotypes in participants with malaria parasitaemia. Additional research on the impact of parasitic infection on the long-term duration of protection from HPV vaccines is warranted.
